# Solution Structural Studies of GTP:Adenosylcobinamide-Phosphateguanylyl Transferase (CobY) from *Methanocaldococcus jannaschii*

**DOI:** 10.1371/journal.pone.0141297

**Published:** 2015-10-29

**Authors:** Kiran K. Singarapu, Michele M. Otte, Marco Tonelli, William M. Westler, Jorge C. Escalante-Semerena, John L. Markley

**Affiliations:** 1 National Magnetic Resonance Facility at Madison and Department of Biochemistry, University of Wisconsin-Madison, Madison, Wisconsin, United States of America; 2 Center for NMR and Structural Chemistry, CSIR-Indian Institute of Chemical Technology, Tarnaka, Hyderabad, Telangana, India; 3 Department of Microbiology, University of Georgia, Athens, Georgia, United States of America; Russian Academy of Sciences, Institute for Biological Instrumentation, RUSSIAN FEDERATION

## Abstract

GTP:adenosylcobinamide-phosphate (AdoCbi-P) guanylyl transferase (CobY) is an enzyme that transfers the GMP moiety of GTP to AdoCbi yielding AdoCbi-GDP in the late steps of the assembly of Ado-cobamides in archaea. The failure of repeated attempts to crystallize ligand-free (apo) CobY prompted us to explore its 3D structure by solution NMR spectroscopy. As reported here, the solution structure has a mixed α/β fold consisting of seven β-strands and five α-helices, which is very similar to a Rossmann fold. Titration of apo-CobY with GTP resulted in large changes in amide proton chemical shifts that indicated major structural perturbations upon complex formation. However, the CobY:GTP complex as followed by ^1^H-^15^N HSQC spectra was found to be unstable over time: GTP hydrolyzed and the protein converted slowly to a species with an NMR spectrum similar to that of apo-CobY. The variant CobY^G153D^, whose GTP complex was studied by X-ray crystallography, yielded NMR spectra similar to those of wild-type CobY in both its apo- state and in complex with GTP. The CobY^G153D^:GTP complex was also found to be unstable over time.

## Introduction

Coenzyme B_12_ (a.k.a. adenosylcobalamin or AdoCbi) is the largest, non-polymeric molecule with biological activity. AdoCbi belongs to the broadly distributed family of cyclic tetrapyrrole molecules known as ‘The Pigments of Life’, which includes hemes, factor F_430_, and chlorophylls [1]. The core ring structure of AdoCbi (a.k.a. the corrin ring) contains a cobalt ion chelated by pyrrolic nitrogens. On the upper (beta) face of the ring, a covalent bond links 5′-deoxyadenosine (Ado) and the Co ion. This unique organometallic bond is critical to the function of the coenzyme. The lower (alpha) face of the ring features a nucleotide loop tethered to a substituent of the ring *via* a phosphodiester bond. Two features unique to the nucleotide loop are the alpha-*N*-glycosidic bond between the base and ribosyl moiety, and the diversity in the base [2]. ‘Cobamide’ is the term used to refer to complete B_12_-like molecules, regardless of their base. The best known cobamide is cobalamin, which contains 5,6-dimethylbenzimidazole as its base.

The assembly of the nucleotide loop evolved differently in bacteria and archaea. In both domains, the pathway starts with the synthesis of AdoCbi-P, which is then converted to AdoCbi-GDP, the so-called activated corrin ring. The difference between the way archaea and bacteria synthesize AdoCbi-GDP lies in the guanylyl transferase that transfers the GMP moiety of GTP to AdoCbi-P. Bacteria use a bi-functional kinase/guanylyl transferase enzyme (CobU, EC 2.7.7.62) [3–5], whilst archaea evolved CobY (E.C. 2.7.7.62), a guanylyl transferase that lacks kinase activity [6]. Crystal structures of CobU in its apo form and in complex with GMP are available (PDB 1C9K [5] and 1CBU [7], respectively). The crystal structure of CobY^G153D^ in complex with GTP is also available (PDB 3RSB) [8], but efforts to crystallize the apo-forms of CobY or CobY^G153D^ were unsuccessful.

Results of biochemical experiments performed during the course of this work revealed that two subunits of apo-CobY bind one GTP molecule with a binding constant of *K*_b_ = 2.0 × 10^−5^ M^-1^ and a dissociation constant of *K*_d_ = 5.0 × 10^−6^ M, but apo-CobY failed to bind GTP analogues, such as GMP-PNP, GMP-PCP or even GDP [9]. CobY binds GTP first before binding AdoCbi-P 200 [9]. The Ado moiety of the corrinoid is required for binding, but the order of binding is clear. The G153D variant of CobY (CobY^G153D^) crystallized in the presence of GTP and led to the determination of the 3D structure of the complex by X-ray crystallography at a resolution of 2.8 Å [8]. Repeated failed attempts to crystallize the apo-CobY protein prompted us to explore solution NMR spectroscopy as a means for determining the structure of apo-CobY and its complex with GTP. To aid in answering how CobY binds GTP and is involved in transferring the GMP moiety to AdoCbi-P, we conducted structural studies using nuclear magnetic resonance (NMR) spectroscopy. We report here the solution structure of apo-CobY, which has allowed comparison with the X-ray structure of CobY^G153D^. We also present NMR studies of CobY^G153D^ and interactions of the proteins with GTP.

## Materials and Methods

### Protein production and sample preparation

[U-^15^N]-CobY, [(U-^13^C, ^15^N]-CobY, and [U-^13^C, ^15^N]-CobY^G153D^ protein samples containing 196 amino acids (residues 1–196) used NMR studies were produced in minimal medium according to the protocol described previously [10], except that *E*. *coli* BL21-CodonPlus^®^ (DE3)-RIL (Stratagene)was used for protein production, and cultures were grown in Erlenmeyer flasks. The *M*.*jannaschiicobY* gene was expressed from plasmid pCobY14 [9]. Proteins were purified as previously reported [9] with the following modifications. Cell-free extract was applied to a 5 mL HiTrap phenyl (high-sub) FF column (GE Healthcare) equilibrated with tris(hydroxymethyl) aminomethane hydrochloride buffer (50 mM Tris-HCI, pH 8.0 at 4°C) containing 55 g/L (NH_4_)_2_S0_4_. Protein was eluted at a flow rate of 5 mL / min with a linear gradient to 100% Tris-HCI buffer. CobY-containing fractions were concentrated and dialyzed against Tris-HCI buffer. Protein purity was assessed as previously reported [9] and found to be >95% homogeneous (data not shown). Ion exchange chromatography was therefore omitted. [U-^13^C, ^15^N]-CobY protein used for structure determination was further dialyzed against 50 mM deuterated Tris buffer (pH 8.0 at 4°C) containing 50mM NaCl, 5 mM dithiothreitol (DTT) and 10 mM MgCl_2_. To prevent bacterial growth, 0.2% NaN_3_ was added to all samples and proteins were stored at 4°C.

### NMR Data Collection and Analysis

All NMR spectra were recorded at the National Magnetic Resonance Facility at Madison (NMRFAM) on Varian VNMRS (600 MHz, 800 MHz and 900 MHz) spectrometers equipped with triple-resonance cryogenic probes. The temperature of the sample was regulated at 40°C. Sequence specific backbone resonance assignments were conducted for CobY using a series of 2D and 3D heteronuclear NMR spectra. NMR data were collected for both CobY containing 2.0 mM [U-^13^C,^15^N] protein dissolved in NMR buffer with 50 mM Tris, 5 mM DTT, 50 mM NaCl, 10 mM MgCl_2_,95% H_2_O, 5% D_2_O. Raw NMR data were processed with NMRPipe [11] and analyzed using the programs XEASY [12] and NMRFAM-SPARKY [13]. 2D ^1^H-^15^N HSQC and 3D HNCO data sets were used to identify the number of spin systems, and these identifications plus 3D HNCACB and 3D CBCA(CO)NH data sets were used as input to the PINE server [14] to determine sequence specific backbone resonance assignments. In addition, backbone resonance assignments were confirmed on the basis of 3D ^15^N-edited ^1^H-^1^H 3D-NOESY. 2D ^1^H-^13^C HSQC, 3D HBHA(CO)NH, 3D HC(CO)NH, 3D C(CO)NH experiments were used to assign the side chain and HB and HA resonances. 3D ^15^N-edited ^1^H-^1^H NOESY (100 ms mixing time), and 3D ^13^C-edited ^1^H-^1^H NOESY (120 ms mixing time) experiments were used to derive the distance constraints to determine the three dimensional structure of protein [15]. Standard pulse sequences were used to record steady state [^1^H]-^15^N NOE and ^15^N relaxation (*T*_1_, *T*_2_) data [16]. To determine the ^15^N *T*_1_ values, multiple interleaved NMR spectra were recorded with relaxation delays of 10, 100, 200, 400, 600, 800, 1000, 1200, and 1400 ms. To determine ^15^N *T*_2_ values, multiple interleaved NMR spectra were recorded with delays of 10, 30, 50, 70, 90, 110, and 150 ms. Relaxation rates were calculated by least-squares fitting of peak heights versus relaxation delay to one single exponential decay by using NMRFAM-SPARKY. The reported error estimates are standard deviations derived from fitting the data. Steady-state [^1^H]-^15^N NOE values were calculated from the ratio of peak heights in a pair of NMR spectra acquired with and without proton saturation. The signal-to-noise ratio in each spectrum was used to estimate the experimental uncertainty.

### Structure calculation and analysis

For the structure calculation, ^15^N resolved ^1^H-^1^H 3D NOESY and ^13^C resolved ^1^H-^1^H 3D NOESY spectra were used to derive the intra molecular distance restraints. TALOS+ software [17] was used to derive backbone dihedral angle restraints φ and ψ from ^1^H, ^15^N, ^13^CA, ^13^CB, ^13^C′chemical shifts. CYANA (version 3.0) [18] was used for automated NOESY peaks assignments and structure calculation. NOESY peaks assigned automatically by CYANA were used as a guide to further refine the structure. Programs MOLMOL [19] and PYMOL [20] were used, respectively, to calculate the root mean square deviation (rmsd) and for graphical analysis. The PSVS server [21] was used to check the quality of the structure.

## Results and Discussion

### Optimization of NMR sample conditions

**Fig 1:**
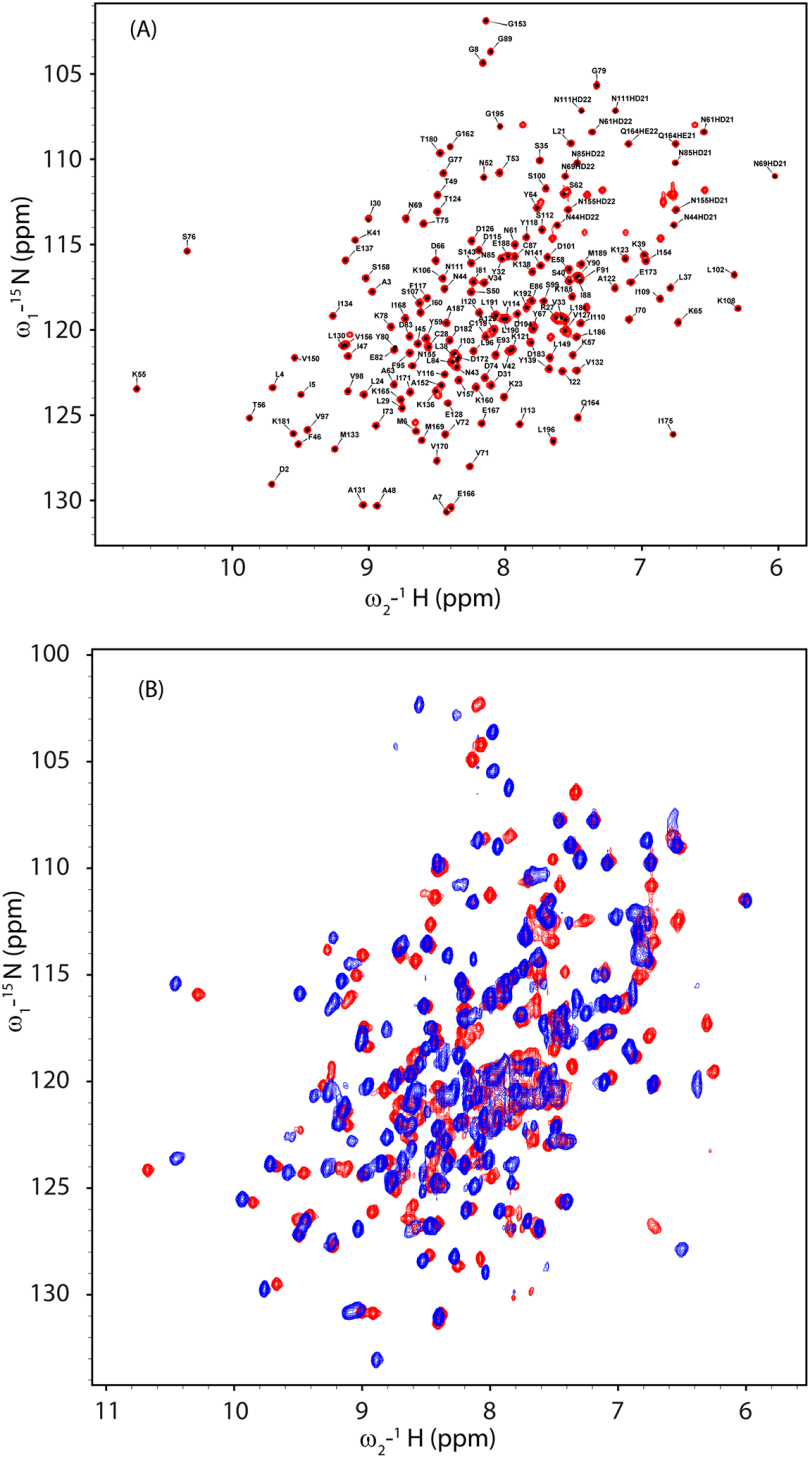
2D ^1^H-^15^N NMR Spectra of CobY and its GTP Complex. (A) ^1^H-^15^N HSQC spectrum of apo-CobY. Amide peaks are labeled with their residue assignments. (B) Overlay of the^1^H-^15^N HSQC spectrum of apo-CobY (red) with that of the CobY:GTP complex (blue).

By optimizing the buffer composition and temperature, we discovered conditions that led to sharp and uniform signals in the ^1^H-^15^N HSQC spectrum (Fig 1A) and good triple-resonance and NOESY data, as needed for a successful structure determination. The final conditions were:2 mM protein in 50 mM TRIS buffer pH 7.0 containing 50 mM NaCl and 10 mMMgCl_2_. Data were collected at 40°C.

### Structure of apo-CobY

The solution NMR structure of apo-CobY was determined from 3246 distance constraints from NOESY spectra and 220 angle constraints derived from chemical shifts by using the TALOS+ program [17]. Two hundred refined structures were generated, and the best 20 conformers, those with lowest energy that showed the fewest constraint violations with CYANA [18], were chosen for additional water bath refinement using PONDEROSA-C/S [22] assisted Xplor-NIH [23].

Statistics for the solution structure (Table 1) are indicative of its high quality. The average number of constraints per residue was 17.6, and, of these, an average of 4.2 per residue were long-range constraints. The root mean square deviation (rmsd) for backbone heavy atoms was< 1.0 Å overall and~0.6 Å for backbone heavy atoms in regular secondary structure. Of the backbone torsion angles, 91% were in the most favored and 8% were in additionally allowed regions of the Ramachandran plot. The PROCHECK [24] Z-scores for backbone / all atoms were−0.04/ −0.71.

**Table 1:** Statistics Describing the NMR Solution Structure of Wild-type apo-CobY.

Constraints	Description	Value
	Conformationally restricting distance constraints (number)	
	Intraresidue [i = j]	1049
	Sequential [(i–j) = 1]	903
	Medium Range [1 < (i–j) ≤ 5]	470
	Long Range [(i–j) > 5]	824
	Total	3246
	Dihedral angle constraints (number)	
	φ	110
	ψ	110
	Constraints per residue (average number)	
	Total	17.6
	Long-range	4.2
	CYANA [18] target function (Å)	6.60+ 0.43
	Average rmsd to the mean coordinates of water refined CNS coordinates (Å)	
	regular secondary structure elements, backbone heavy	0.62±0.08 ^a^
	regular secondary structure elements, all heavy atoms	0.94+0.08 ^b^
	backbone heavy atoms	0.66±0.07 ^b^
	all heavy atoms	1.05±0.09 ^b^
Validation parameters	Description	
	PROCHECK [24] rawscore (φ and Ψ/all dihedral angles)	-0.7/-0.12 ^c^
	PROCHECK Z-scores (φ and Ψ/all dihedral angles)	0.04/-0.71 ^c^
	MOLPROBITY [25] raw score/Z-score	34.76/-4.44 ^c^
	Ramachandran plot summary: ordered residue ranges (%)	
	most favored regions	91.5
	additionally allowed regions	8.2
	generously allowed regions	0.2
	Average number of distance constraint violations per CYANA conformer	
	0.2–0.5 Å	0.0
	> 0.5 Å	0.0
	Average number of dihedral-angle constraint violations per CYANA conformer	0.0

^a^ Residues:2–7 (β1), 22–24, 27–29, 29–39(α1), 44–49 (β2), 54–64 (α2), 70–74 (β3), 80–90 (α3), 95–99(β4), 101–104, 107–123 (α4), 129–135(β5), 149–157(β6), 166–170(β7), 175–177, 181–195 (α5).

^b^ Residues: 2–7, 21–135, 148–195.

^c^ Residues: 2–7, 20–25, 27–102, 106–139, 149–152, 154–160, 163–176, 179–194.

**Fig 2:**
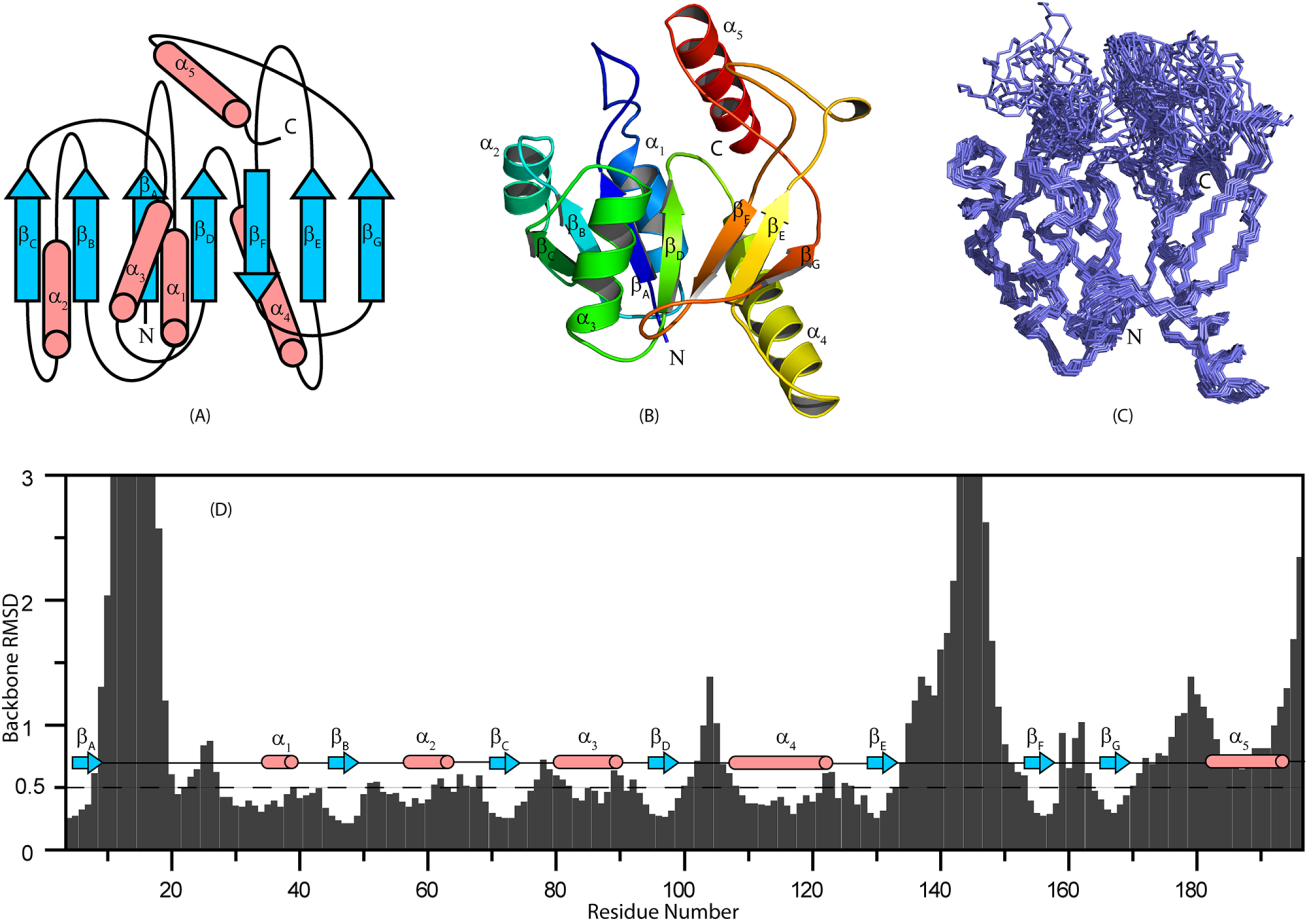
Three-dimensional Solution Structure of apo-free CobY. (A) Secondary structure topology representing seven β-stands and five α-helices. (B) Ribbon diagram of the lowest energy conformer of apo-CobY. (C) Overlay of the regular secondary structure of the 20 lowest energy conformers that showed the fewest violations in CYANA (N- and C-terminals are labeled). (D) The rmsd of the backbone atoms plotted against the corresponding amino acid number. Residues 10–20 and 135–150 displayed the highest rmsd values. Also shown are the positions of the α-helices and β-strands.

The structure consists of a mixed α/β fold (Fig 2). The seven β-strands (A-G) consist of residues β_A_(D2−M6), β_B_(N44−T49), β_C_(I70−D74), β_D_(F95−S99), β_E_(A129−M133), β_F_(P151−V157), and β_G_(E167−V170). The five α-helices (I-V) consist of residues α_I_(L29−K39), α_II_(P54−Y64), α_III_(Y80−Y90), α_IV_(K108−K123), and α_V_(T180−K192). The orientations of the β-strands make up a twisted β-sheet (Fig 2A, 2B, and 2C); six of the seven β-strands are arranged in parallel fashion: β_C_(↑) β_B_(↑) β_A_ (↑) β_D_(↑) β_F_(↓) β_E_(↑) β_G_(↑). In addition, a short and stable β-hairpin is located between residues I22 and L29, and a short anti-parallel β-sheet-like structure is formed by residues D101-N104 and I175-N177. Four α-helices (I, II, IV, and V) are arranged on one side of the β-sheet, whereas one α-helix (IV) is on the other side of the β-sheet. α-Helix I is in contact with β-strands A and B, whereas α-helix II is in contact with β-strands B and C. α-helices I and II also contact one other; α-helix III contacts half of the β-sheet (β-strands C, B, A, D, and F); and α- helix IV contacts the other side of the β-sheet (β-strands A, D, F, E, and G). The loops connecting the secondary structural elements (α_1_−β_A_, α_4_−β_D_, β_E_−β_F_, and β_G_−α_5_) are highly flexible and unstructured. Resonance assignments could not be obtained for residues in some of these loops because of exchange broadening, which led to the disappearance of the amide peaks. The backbone rmsd plotted against the amino acid sequence (Fig 2D) shows that the polypeptide chain is flexible between residues 8–20 and133–153. The C-terminal helix is also relatively dynamic as determined from heteronuclear NOE values. The coordinates were deposited in the Protein Data Bank (PDB) with accession code 2MZB, and the chemical shifts were deposited in Biological Magnetic Resonance Data Bank (BMRB) with accession code 25482.

### Structural homologues of apo-CobY

**Fig 3:**
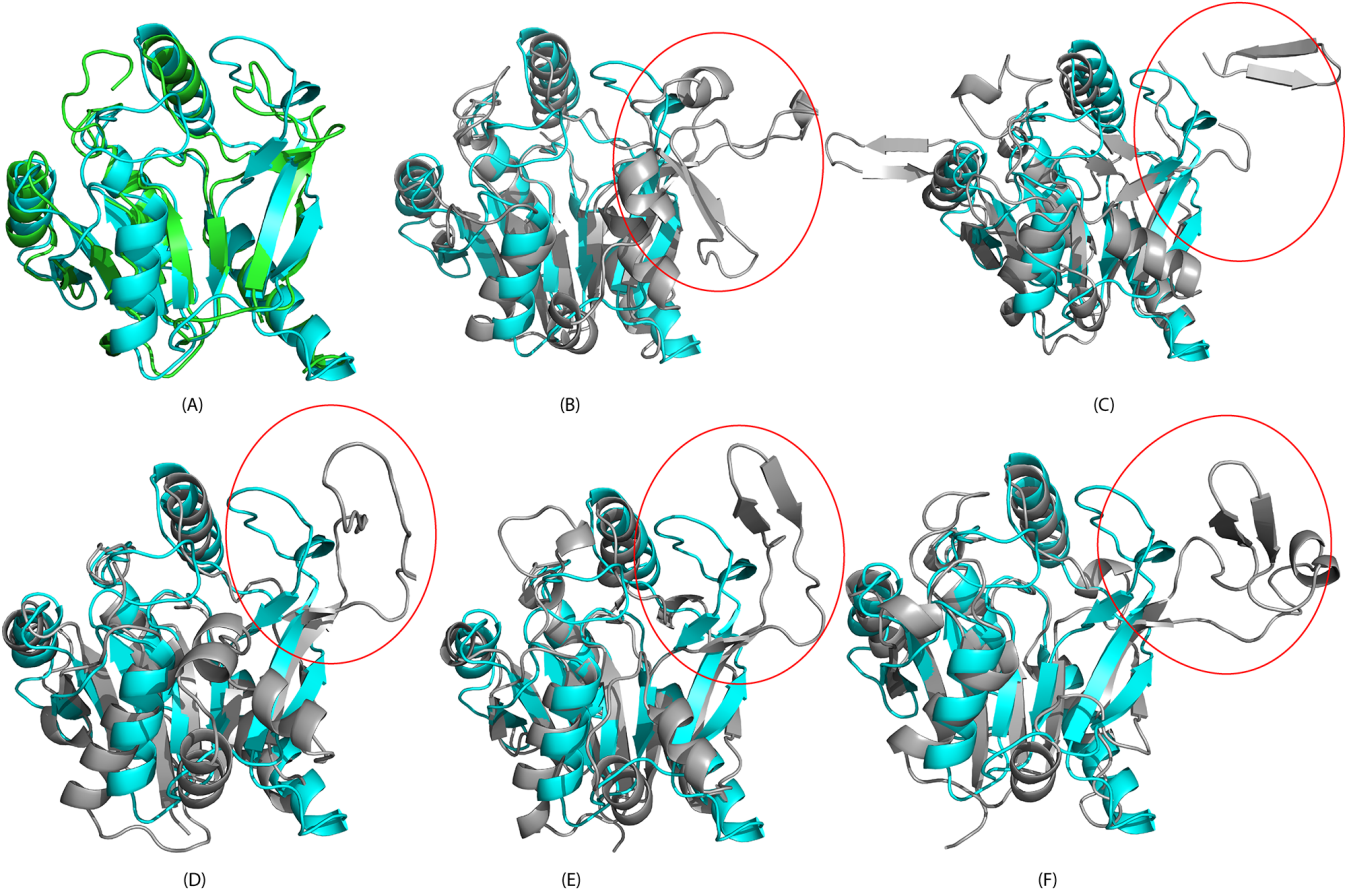
Overlay of the Ribbon Structure apo-CobY (cyan) with those of Structurally Similar Proteins. (A) (green) X-ray structure of the CobY^G153D^:GTP complex (PDB 3RSB) (87% structural overlap with rmsd 1.82 Å; fragment/topology score 0.95/0.94 with match size 113). (B) (gray) X-ray structure of CMP:2-keto-3-deoxy-manno-octonic acid synthetase (PDB 1H7F).(75.5% structural overlap with rmsd 1.84 Å; fragment/topology score 0.93/1.0 with match size 148). (C) (gray) X-ray structure of α-D-glucose-1-phosphate cytidylyl transferase (PDB 1WVC) (75.5% structural overlap with rmsd 2.15 Å; fragment/topology score 0.86/1.0 with match size 148). (D) (gray) X-ray structure of acytidylyl transferase (PDB 2VSI) (74.5% structural overlap with rmsd 1.98 Å; fragment/topology score 0.91/1.0 with match size 146). (E) X-ray structure of 2-C-methyl-D-erythritol 4-phosphate cytidylyl transferase(PDB 1VPA) (73% structural overlap with rmsd 1.86 Å; fragment/topology score 0.91/1.0 with match size 143). (F) X-ray structure of CMP-acylneuraminate synthetase (PDB 1EYR) (70% structural overlap with rmsd 1.95 Å; fragment/topology score 0.88/1.0 with match size 137). PYMOL [20] was used to generate the structures, and the CLICK [33] program was used to carry out the pairwise alignments.

**Fig 4:**
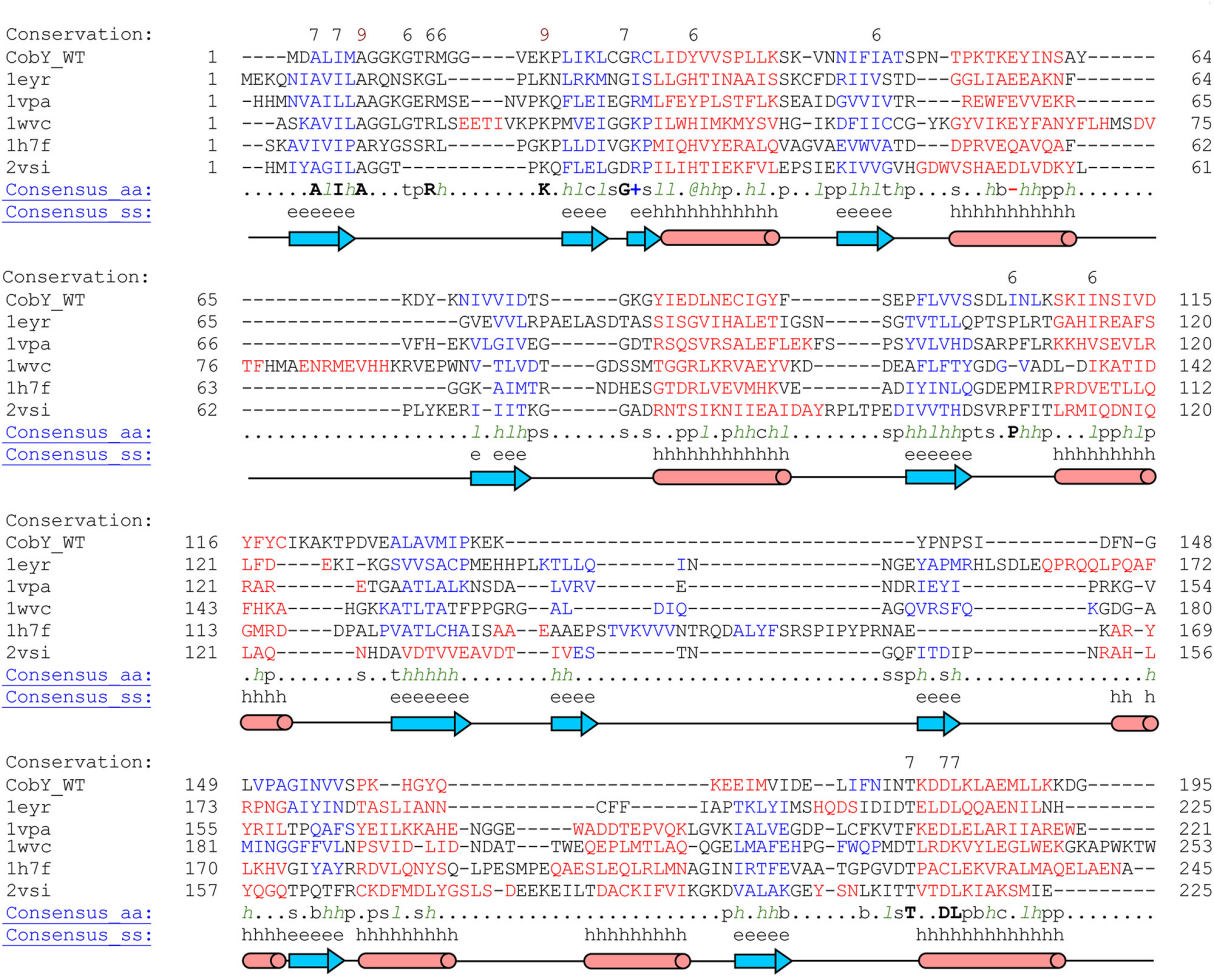
Structure-based Multiple Sequence Alignment of Wild-type apo-CobY with the Five Most Structurally Similar Proteins. The alignment, which was carried out using the PROMALS3D program [32], shows substantial sequence alignment of secondary structural elements derived from the 3D structures: (blue) α-helix; (red) β-strand.

We used the software programs DALI [26] and ProFunc [27] to search for structural homologues of apo-CobY. The five most similar structures, all determined by X-ray crystallography, contained mononucleotide binding domains with a canonical Rossmann fold (Fig 3). Cytidinyl monophosphate2-keto-3-deoxy-manno-octonic acid synthetase (CMP:Kdo) from *Escherichia coli* (PDB 1H7F, Z-score 15.8, rmsd 2.6 Å, seq ID 14%) is involved in the synthesis of lipopolysaccharides that are toxic to Gram-negative bacteria [28]. Glucose-1-phosphate cytidylyl transferase from *Salmonella typhi* (PDB 1WVC, Z-score 15.2, rmsd 3.3 Å, seq ID 18%) catalyzes the transfer of a CMP moiety from CTP to glucose 1-phosphate [29]. Cytidine transferase from *Streptococcus pneumonia* (PDB 2VSI, Z-score 15.6, rmsd 3.0 Å, seq ID 13%) is involved in the synthesis of cytidine-5'-diphosphate (CDP)-ribitol from ribitol 5-phosphate and CTP [30]. 2-C-methyl-D-erythritol 4-phosphate cytidylyl transferase from *Thermotoga maritima* (PDB 1VPA, Z-score 15.3, rmsd 3.1 Å, seq ID 18%) catalyzes the formation of 4-diphosphocytidyl-2-C-methyl-D-erythritol from CTP and 2-C-methyl-D-erythritol 4-phosphate. N-acylneuraminate cytidylyl transferase from *Neisseria meningitides* (PDB 1EYR, Z-score 15.3, rmsd 3.0 Å, seq ID 17%) catalyzes the reaction of CTP and N-acylneuraminate to form CMP-N-acylneuraminate and bisphosphate [31]. A structure-based multiple sequence alignment performed by PROMALS3D [32] revealed several highly-conserved residues (Fig 4): A3, I5, A7, R13, K19, G26, (K/R)27, (D/E)58, T180, D183, and L184. These residues appear to play important roles in nucleotide binding. In particular, residues R13 and K19 directly coordinate the phosphate group of GTP in the X-ray structure of CobY^G153D^ [8].

All these structures share < 20% sequence identity with CobY and additionally contain HTH motifs and/or a β-hairpin structure that facilitates the formation of homodimers (Fig 4).

### Dynamics of ligand free CobY

**Fig 5:**
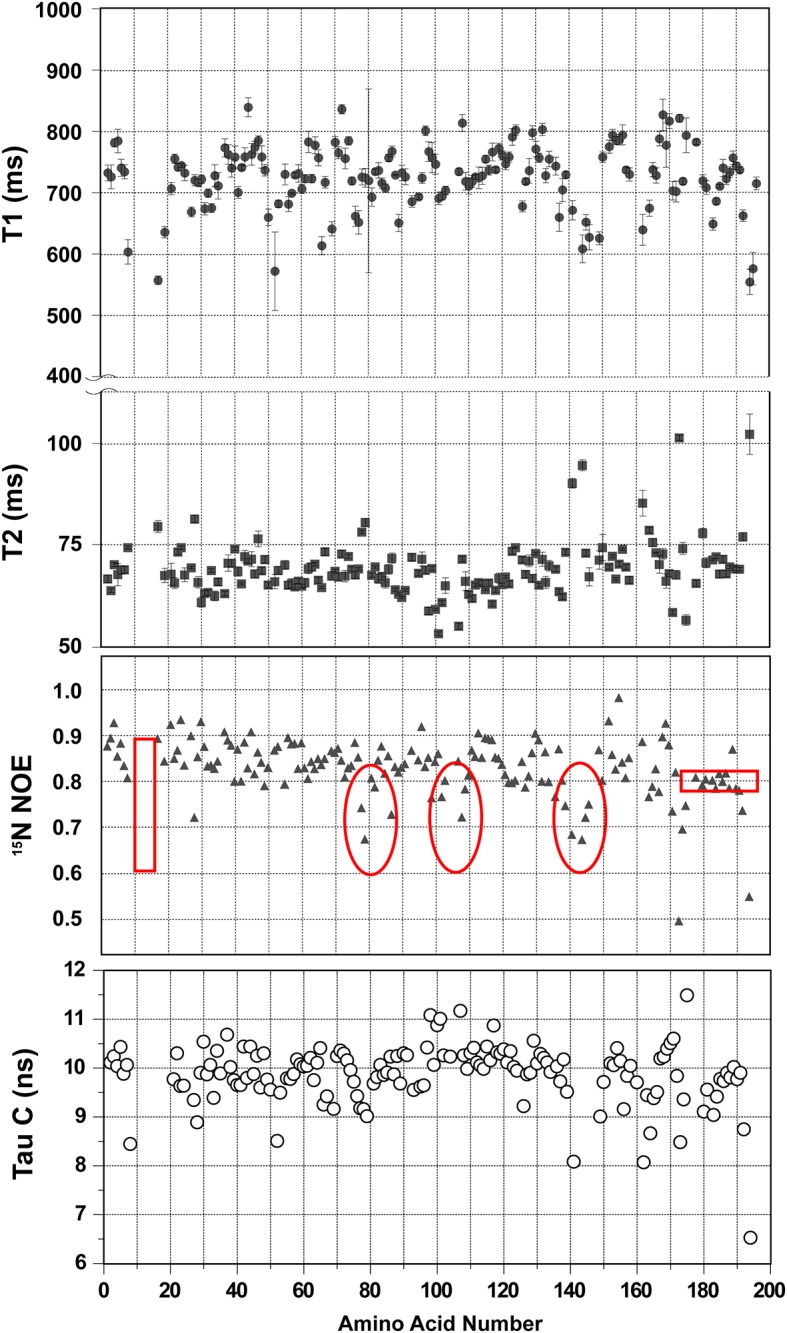
Dynamics of apo-CobY as Represented by Residue-specific Backbone Amide Spin-lattice (*T*_1_) and Spin-spin (*T*_2_) Relaxation Times, Heteronuclear NOE (^15^N NOE) Values, and Correlation Times. The flexible regions of the polypeptide chain are circled in red; the vertical red box represents missing resonances from residues that are assumed to be flexible; and the horizontal red box indicates the C-terminal helix, which appears to be more flexible than other secondary features as indicated by the smaller ^15^N NOE values. The τ_c_ values were calculated from measured *T*_1_ and *T*_2_ values by using the formula, τ_c_ = 1/4πν_N_ (√6(*T*1/*T*2) − 7). The average τ_c_ value (9.9 ± 0.7 ns) indicates that CobY is monomeric in solution under the NMR sample conditions.

One of the advantages of NMR spectroscopy over X-ray crystallography is its ability to provide information about the dynamic properties of proteins in solution. For CobY we accomplished this by measuring nitrogen spin-lattice (*T*_1_) and spin-spin (*T*_2_) relaxation times and heteronuclear NOEs (^15^N NOEs) for backbone amide resonances. As shown in Fig 5, we found that the relaxation parameters are fairly similar throughout the polypeptide chain, indicating uniform overall protein dynamics. The only exceptions were inflexible loops connecting regular secondary structure elements, in particularα_1_-β_A_, α_4_-β_D_, β_E_-β_F_, and β_G_-α_5_; these regions yielded weak electron density in the X-ray studies of CobY. The average *T*_1_ (~750 ms) and *T*_2_ (~70 ms)values are those expected for a monomeric protein of ~23 kDa and are consistent with the 3D NMR solution structure. In addition, the rotational correlation times (τ_c_) of the amide protons calculated from *T*_1_ and *T*_2_ revealed an average τ_c_ = 9.9 ± 0.7 ns (Fig 5) consistent with monomeric protein in solution. By contrast, the X-ray structure of the CobY^G153D^:GTP complex was modeled as a weak dimer [8].

### Comparison of the NMR structure of apo-CobY and the X-ray structure of the CobY^G153D^:GTP complex

**Fig 6:**
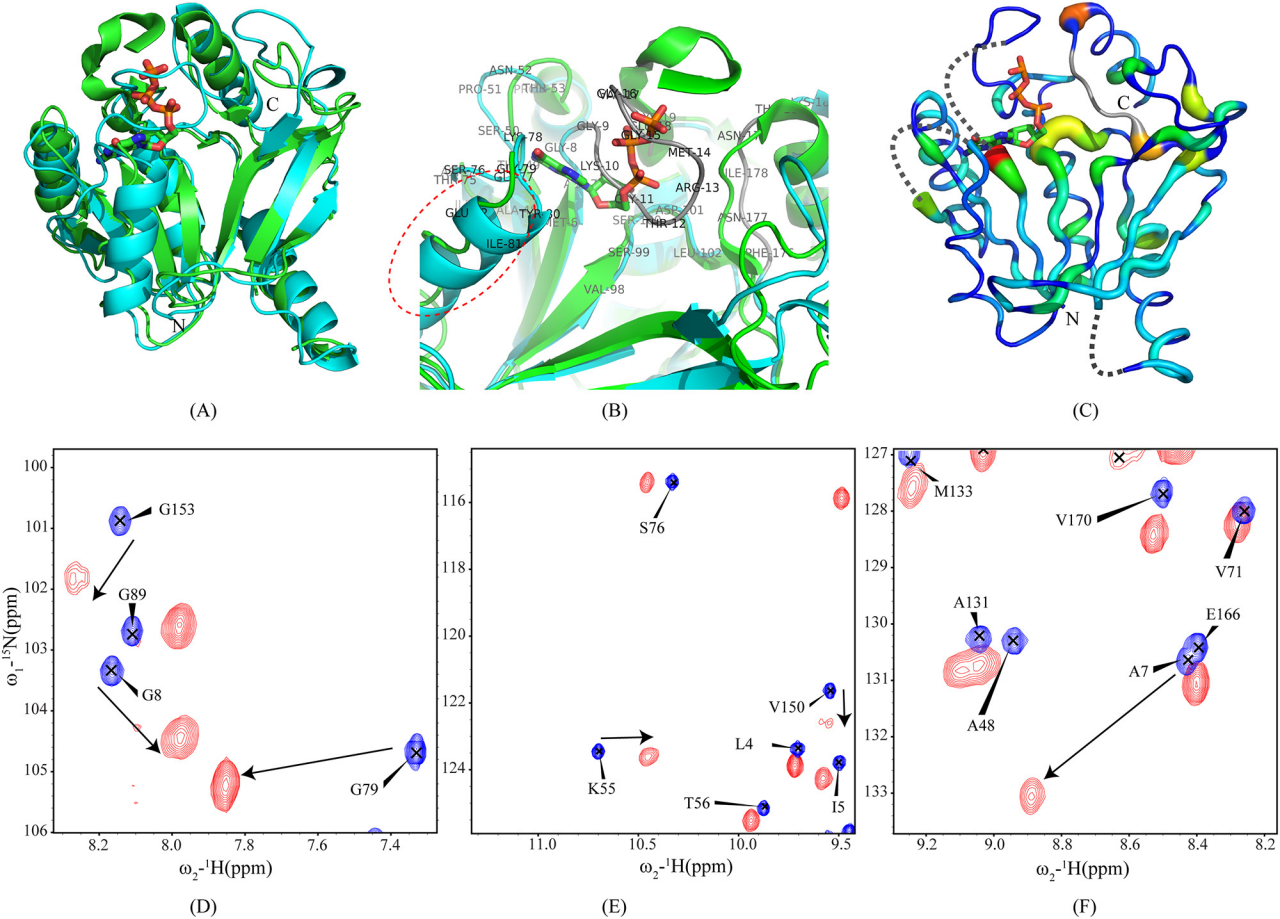
Comparison of Structures of apo-CobY and CobY^G153D^:GTP Complex and Comparison of ^1^H-^15^N HSQC Spectra of CobY and CobY:GTP. (A) Superposition of the 3D structures of apo-CobY determined by NMR (cyan) and CobY^G153D^:GTP determined by X-ray crystallography (green) with the GTP displayed as a stick model. (B) Expansion of the overlaid structures showing the GTP binding site. Residues perturbed upon complex formation with GTP are annotated. Whereas α-helix-III (circled in red) is well defined in the structure of apo-CobY, it is poorly resolved in the X-ray structure of the complex. (C) Weighted rmsd chemical shift differences ([0.5[Δδ(^1^H^N^)^2^+ (0.2Δδ(^15^N))^2^]]^1/2^) between apo-CobY and CobY:GTP mapped onto the X-ray structure of CobY^G153D^:GTP. The magnitude of the shift is coded by a spectrum with red largest shift and blue small or no shift. Residues not detected or assigned in both NMR in the spectra compared are shown in gray. The dotted regions of the polypeptide chain represent ones for which no electron density was detected (D-F) Overlays of three regions of the ^15^N HSQC spectra of CobY (blue) and CobY:GTP (red). The cross peaks from CobY are annotated with their assignments, and the arrows indicate the changes in peak positions upon complex formation with GTP.

The solution structure of apo-CobY and the crystal structure of CobY^G153D^:GTP complex(PDB 3RSB) [8] exhibit similar 3D folds (structures superimposed in Fig 6A). The elements of regular secondary structure (consisting of 67 amino acid residues) superimposed with an average rmsd = 0.83Å. The X-ray structure has a relatively low resolution (2.8 Å), and electron density was not identified for residues 8–11, 74–81, 126–127, and 192–196. Perhaps the largest structural differences are in α-helix-III, which is structured in the NMR solution structure but unstructured in the X-ray structure (Fig 6B). Although isothermal titration calorimetry (ITC) studies suggested one GTP molecule per two units of CobY [9], the X-ray structure of the CobY^G153D^:GTP complex was modeled as a dimer with one GTP molecule bound to each subunit. The ITC results were obtained with active enzyme that may have been turning over during the experiment. Our NMR experiments with both CobY and CobY^G153D^ are consistent with a 1:1 complex.

### Complex formation of CobY with GTP

To probe the effect of added GTP on apo-CobY, we titrated a sample of ^15^N-labeled apo-CobY with GTP and followed the chemical shifts in a series of ^1^H-^15^NHSQC spectra. Several amide cross peaks exhibited large perturbations (Fig 6D–6F), indicating that significant conformational changes accompanied the formation of the GTP complex. ^1^H-^15^N HSQC spectra acquired following the addition sub-stoichiometric amounts of GTP (not shown), exhibited two sets of peaks, one corresponding to free CobY and one to the CobY:GTP complex; this indicates a slow off rate for GTP dissociation.

Two factors appear to be responsible for these chemical shift changes: (i) GTP-induced ordering of the binding domain and (ii) electrostatic interactions between the ligand and protein backbone. In the X-ray structure of the CobY^G153D^: GTP complex, the tri-phosphate group of GTP is in the proximity of the region of the protein (residues 10–18) that appears disordered in the apo-enzyme both in X-ray crystal data, which lacked electron density for these residues, and in the solution spectra, which lacked signals from these residues as attributed to exchange broadening. The overlaid expansions of three regions of the ^15^N HSQC spectra of CobY and CobY:GTP complex (Fig 6D–6F) indicate that the amide protons of A7, G8, K55, G79, G153 shift significantly upon GTP binding. The weighted chemical shift perturbations (CSPs) mapped on 3D structure of CobY^G153D^ (Fig 6C) show that some are close to the GTP binding site and others are distant.

### Comparison of CobT and CobY^G153D^

**Fig 7:**
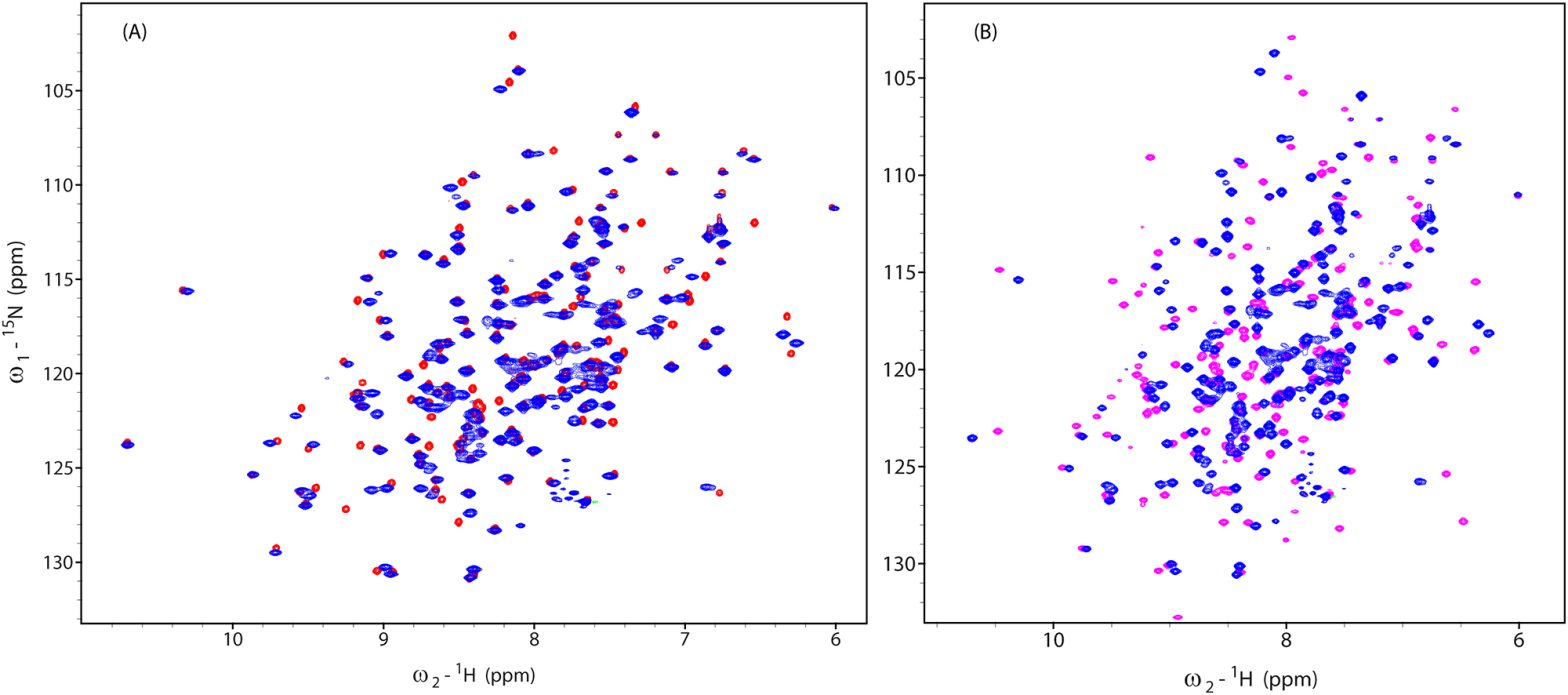
Comparison of ^1^H-^15^N HSQC Spectra of CobY and CobYG153D. (A) Spectrum of apo-CobY (red) overlaid with that of apo-CobYG153D(blue). (B) Spectrum of apo-CobYG153D (blue) overlaid with that of the CobYG153D:GTP complex (purple).

Comparison of the ^1^H-^15^N HSQC spectra of [U-^15^N]-CobY and [U-^15^N]-CobY^G153D^ (Fig 7A) indicates that the chemical shift differences are small. The sample of ^15^N-labeled CobY^G153D^ was saturated with GTP, and the resulting spectrum was compared to that of apo-CobY^G153D^ (Fig 7B). The chemical shift perturbations upon GTP binding are very similar to those observed with wild-type CobY (Fig 1B).

**Fig 8:**
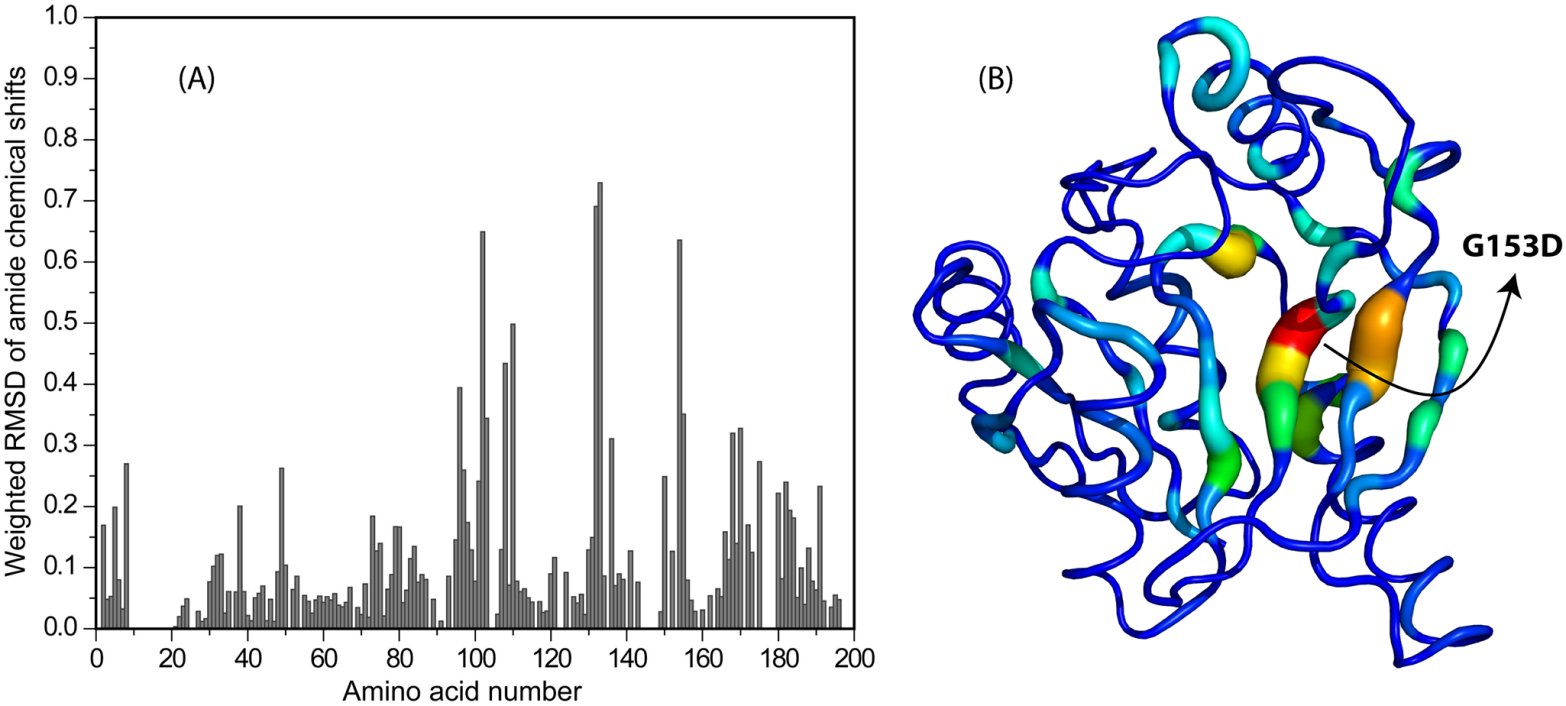
Representation of Differences in the Chemical Shifts of CobY and CobY^G153D^. (A) Weighted rmsd of amide proton chemical shift differences ([0.5[Δδ(^1^H^N^)^2^+ (0.2Δδ(^15^N))^2^]]^1/2^) plotted as a function of the amino acid residue number. (B) Mapping of the weighted rmsd chemical shift differences onto the 3D NMR structure of CobY. Color code: (red, yellow, green, cyan) spectrum of chemical shift differences from largest to small; (blue) no significant chemical shift difference.

The differences in the chemical shifts of CobY and CobY^G153D^ are plotted as a function of residue number in Fig 8A and are mapped onto the 3D structure of the protein in Fig 8B. As expected, atoms in residues near residue 153 (the substitution site) exhibit the largest chemical shift differences.

The GTP complexes with wild-type CobY and CobY^G153D^ proved to be unstable in solution. NMR spectra taken over time (not shown) indicated each complex converted to an unknown intermediate state over a period of about 24 hours (most probably the “switch-off” GDP complex) and then converted over a period of days to species with spectra resembling those of the apo-proteins. Repeated attempts to make a stable GTP-CobY complex by reducing the temperature and saturating with GTP were unsuccessful. The instability of the complexes prevented us from determining their solution structures.

## Conclusions

Solution NMR studies of apo-CobY yielded a 3Dstructure of high quality with a fold is similar to that of the low resolution X-ray structure of the CobY^G153D^:GTP complex [8]. We found CobY to be monomeric in solution in both its apo- and GTP-bound forms, whereas the X-ray structure of the CobY^G153D^:GTP complex was modeled as a homodimer. Other differences may reflect problems in tracing the chain in the X-ray map.

It is known that complexes of GTPases with GTP are conformationally flexible to allow for the conversion of GTP to GDP and transfer of the phosphate group. The proposed two-state mechanism has been extensively studied for small GTPases, such as Ras, RhoA, and Sec4 [34]. The active “switch-on” state has GTP bound, whereas the inactive “switch-off” state has GDP bound. Titration studies of CobY followed by NMR spectroscopy revealed that CobY forms a tight 1:1 complex with GTP. However, the complex was found to degrade over time, which prevented the determination of its solution structure.

## Abbreviations

Apo-CobY: wild-type, ligand-free CobY
CobY^G153D^: G153D variant of CobY
BMRB: Biological Magnetic Resonance Data Bank
DTT: dithiothreitol
HSQC: heteronuclear single-quantum coherence
NMR: nuclear magnetic resonance
NOE: nuclear Overhauser enhancement
NOESY: NOE spectroscopy
PDB: Protein Data Bank
rmsd: root mean square deviation
τ_c_: rotational correlation time
TALOS: torsion angle likelihood obtained from shifts
